# Growth in extremely preterm children born in England in 1995 and 2006: the EPICure studies

**DOI:** 10.1136/archdischild-2020-321107

**Published:** 2021-07-13

**Authors:** Yanyan Ni, Rebecca Lancaster, Emmi Suonpera, Marialivia Bernardi, Amanda Fahy, Jennifer Larsen, Jayne Trickett, John R Hurst, Dieter Wolke, Samantha Johnson, Neil Marlow

**Affiliations:** 1 EGA Institute for Women's Health, University College London, London, UK; 2 Department of Psychology, University of Warwick, Coventry, UK; 3 Department of Health Sciences, University of Leicester, Leicester, UK; 4 UCL Respiratory, University College London, London, UK; 5 Division of Health Sciences, Warwick Medical School, University of Warwick, Coventry, UK

**Keywords:** growth, neonatology

## Abstract

**Objectives:**

To determine growth outcomes at 11 years of age in children born <27 weeks of gestation in England in 2006 (EPICure2) and to compare growth from birth to 11 years of age for births<26 weeks with those in England in 1995 (EPICure).

**Methods:**

200 EPICure2 children assessed at 11 years alongside 143 term-born controls. Growth measures from birth to 11 years were compared for births<26 weeks between EPICure2 (n=112) and EPICure (n=176). Growth parameter z-scores were derived from 1990 UK standards.

**Results:**

Among EPICure2 children, mean z-scores for height and weight were close to the population standards (0.08 and 0.18 SD, respectively) but significantly below those of controls: difference in mean (Δ) z-scores for weight −0.42 SD (95% CI −0.68 to –0.17), for height −0.45 SD (−0.70 to –0.20) and for head circumference (HC) −1.05 SD (−1.35 to –0.75); mean body mass index (BMI) z-score in EPICure2 children was 0.18 SD, not significantly different from controls (0.43 SD, p=0.065). Compared with EPICure, EPICure2 children born <26 weeks at 11 years had higher z-scores for weight (Δ 0.72 (0.47, 0.96)), height (Δ 0.55 (0.29, 0.81)) and BMI (Δ 0.56 (0.24, 0.87)), which were not fully explained by perinatal/demographic differences between eras. Weight catch-up was greater from term-age to 2.5/3 years in EPICure2 than in EPICure (1.25 SD vs 0.53 SD; p<0.001). Poor HC growth was observed in EPICure2, unchanged from EPICure.

**Conclusions:**

Since 1995, childhood growth in weight, height and BMI have improved for births <26 weeks of gestation, but there was no improvement in head growth.

## Abstract (precis)

This paper describes the growth to age 11 of a sample of the EPICure 2 2006 preterm birth cohort, compared to healthy same age controls and to the 1995 EPICure cohort. Since 1995, childhood growth in weight, height and BMI have improved for births <26 weeks of gestation, but there was no improvement in head growth.

**What is already known on this topic?**

- Survival for extremely preterm babies has improved since 1995 in the UK.
- There was no improvement in physical growth from birth to full-term age between babies born <26 weeks of gestation in 1995 (EPICure) and 2006 (EPICure2).
- It is unclear whether long-term growth outcomes have changed.

**What this study adds?**

- Among EPICure2 children, mean z-scores for height and weight at 11 years were close to the UK 1990 population standards but significantly below those of controls.
- Despite advancing perinatal care since 1995, head growth has not improved for children born <26 weeks’ gestation in 2006.
- Improvements were observed in weight, height and body mass index during infancy and middle childhood for births <26 weeks of gestation in 2006.

## Introduction

Poor growth attainment from infancy to early adulthood has been reported for children born extremely preterm (EP) in the 1990s in comparison with term-born peers.^1–4^ However, information on growth outcomes is lacking for EP children born in the 2000s. Survival for EP babies has improved since 1995 in England^5 6^ and in other countries.^7–9^ Despite reports of trends towards improved neurodevelopmental outcomes during infancy and early childhood for EP babies born after the 1990s,^10 11^ as yet there is no evidence that early improvements are sustained in middle childhood. There are few reports of physical growth over time. We previously compared weight and head circumference (HC) for births <26 weeks’ gestation in England and found no improvement in somatic or head growth from birth to the expected date of delivery (EDD) between babies born in 1995 and 2006.^5^ Less is known about whether later growth parameters have changed since 1995.

The aims of this paper were: (1) to investigate growth measures at 11 years of age for children born <27 weeks’ gestation in England in 2006 in comparison with term-born controls, as part of a longitudinal cohort study (EPICure2 study) and (2) to compare growth measures from birth to 11 years of age for children born <26 weeks of gestation with those of babies born in England in 1995 who were followed up as part of the original EPICure study.^4^ We expected to see improved growth for EP children in EPICure2, but it was unclear whether this extended to improvement in head growth, as we have previously reported no change in neurodevelopmental outcomes between the two cohorts.^12^

## Methods

### Participants

The EPICure2 study comprises all EP births<27 weeks of gestation in England during 2006.^5^ A total of 1041 babies survived to discharge, and children were followed up at age 3 and 11 years. Ten deaths occurred between discharge and the 3-year assessment. Recruitment and assessment at 3 years have been described previously: 576 children were assessed.^6^ At 11 years, invitations to participate in the study were sent to a sample of parents of 482 children comprising births in 17 clinical neonatal networks in England. As part of the study design, a contemporary comparison group was recruited. For each EP child in mainstream school, up to three term-born controls were recruited from classmates of the same age (±3 months) and sex. Where it proved impossible to gain access to the school or at a parent’s request, children were assessed at home. In these cases, a term-born child was identified by the parent of the EP child where possible and was invited to participate in the study. For children attending special educational needs schools (n=22), controls were not recruited. Assessors were not informed of the child’s birth status. Further information on study procedure is provided in the online supplemental appendix. The EPICure study comprised all births <26 weeks of gestation in the UK and Ireland during 1995. Recruitment of the cohort has been described previously;^13^ 315 children survived to discharge and were invited for follow-up assessments at age 2.5, 6, 11 and 19 years.

10.1136/fetalneonatal-2020-321107.supp1 Supplementary data

### Measures

In EPICure2, growth data were collected at birth and EDD (weight and HC), and at age 3 and 11 years (weight, height, HC and body mass index (BMI)). In EPICure, growth data were available at birth and EDD (weight and HC), and at age 2.5, 6 and 11 years (weight, height, HC and BMI).^1 3 4^ For both cohorts, SD or ‘z’ scores for growth measures were calculated based on the British 1990 growth reference adjusting for sex and age.^14^ Classification for child overweight and thinness was defined according to international standards using age and sex specific BMI cut-oﬀ points.^14^ Birth weight z-scores were derived from the original EPICure cohort data which were complete population samples from which the study children are drawn.^15^ The Index of Multiple Deprivation 2015 (IMD), the version closest to assessment dates, was used as a measure of socio-economic status at 11 years in EPICure2 and was obtained using postcode of parent’s residence at the time of the assessment.^16^ IMD ranks were used to derive deciles based on the English population with Decile 1 (most deprived) to Decile 10 (least deprived). The IMD 2007 version was used at 11-year assessment in EPICure.

### Analysis

Analyses were performed in STATA V.15.1. For EPICure2, summary data on the neonatal variables were presented for those formally assessed at 11 years and those not assessed. Linear regression was used to estimate difference in means (Δ) and 95% CI for growth measure z-scores between EP children and controls at 11 years, as well as to investigate impacts of gestational age in weeks and days on growth. For the comparison between EPICure and EPICure2, we only included births <26 weeks’ gestation to women resident in England at birth. We compared growth measures at comparable ages between the two cohorts and examined the trend over time using repeated measures mixed models. We estimated relative risk ratios of child overweight/obesity and thinness for EPICure2 compared with EPICure using multinomial logistic regression models. We further adjusted for perinatal and demographic differences between eras in the above models. We performed multiple imputation^17^ as a sensitivity analysis to account for missing data (online supplemental table S1). Missing data were imputed by chained equations using the STATA ‘MI’ procedure. Imputation models were based on the missing at random assumption and 20 imputed datasets were created. Original and imputed results were similar (online supplemental table S2), so we only report the original results.

## Results

### Outcomes for births <27 weeks of gestation in EPICure2

#### Participants and attrition

Of the 482 EPICure2 invitees at 11 years, 220 gave consent to participate. Due to difficulties in scheduling, we evaluated 200 (19%) of the 1031 EPICure2 EP children known to be alive at 3 years. Baseline information for the assessed children was compared with the non-evaluated sample (table 1). Assessed EP children were more likely to be born to mothers of older age and to have received breast milk at discharge; there was a higher proportion of South Asian children and lower proportions of White and Black children among those assessed. The assessed sample is representative of the original cohort in terms of gestational age, birth weight, sex and other birth characteristics. In the EP group, mean (SD) age at assessment was 11.8 (0.5) years; mean IMD (SD) was 5.2 (2.8); 50% were boys. These characteristics did not significantly differ from 143 controls.

**Table 1:** Comparison of perinatal and childhood variables between children formally evaluated at 11 years and non-responders in 2006 birth cohort

Variables		Children assessed at 11 years (n=200)	Children not assessed at 11 years (n=831)	P value
Gestational age				
22 weeks	% (n/N)	0.0% (0/200)	0.4% (3/831)	0.536
23 weeks	% (n/N)	7.5% (15/200)	5.8% (48/831)	
24 weeks	% (n/N)	14.0% (28/200)	17.9% (149/831)	
25 weeks	% (n/N)	34.5% (69/200)	32.7% (272/831)	
26 weeks	% (n/N)	44.0% (88/200)	43.2% (359/831)	
Birth weight (g)	Mean (SD)	810.2 (147.5) (n=200)	794.5 (149.0) (n=831)	0.181
Birth weight z scores	Mean (SD)	−0.2 (0.8) (n=200)	−0.3 (0.8) (n=828)	0.163
Male sex	% (n/N)	50.0% (100/200)	48.0% (399/831)	0.614
Multiple birth	% (n/N)	24.5% (49/200)	24.0% (202/841)	0.886
Ethnicity				
White	% (n/N)	59.6% (118/198)	67.0% (549/819)	0.009
Asian	% (n/N)	16.2% (32/198)	8.7% (71/819)	
Black	% (n/N)	18.7% (37/198)	20.5% (168/819)	
Other	% (n/N)	5.6% (11/198)	3.8% (31/819)	
Maternal age at delivery	Mean (SD)	30.7 (6.0) (n=200)	28.7 (6.6) (n=830)	<0.001
IMD at birth	Mean (SD)	4.5 (2.7) (n=198)	4.3 (2.9) (n=824)	0.287
Maternal height	Mean (SD)	163.0 (6.8) (n=179)	162.7 (7.0) (n=652)	0.570
Maternal weight	Mean (SD)	69.1 (16.3) (n=172)	68.2 (16.5) (n=640)	0.572
Breast milk at any time	% (n/N)	98.5% (197/200)	95.7% (794/830)	0.059
Breast milk at discharge	% (n/N)	56.0% (112/200)	39.3% (325/828)	<0.001
No previous birth ≥24 weeks	% (n/N)	54.8% (109/199)	52.5% (434/827)	0.560
Worst cerebral ultrasound scan	% (n/N)	18.6% (37/199)	21.6% (179/827)	0.343
Antenatal systemic steroids	% (n/N)	86.0% (172/200)	87.7% (718/819)	0.525
Postnatal systemic steroids	% (n/N)	16.5% (33/200)	15.0% (125/831)	0.607
Cervical suture	% (n/N)	8.0% (16/199)	5.8% (48/830)	0.236
Parenteral nutrition	% (n/N)	100.0% (200/200)	99.9% (830/831)	0.624
Days to parenteral nutrition	Median (range)	1 (0–6) (n=198)	2 (0–14) (n=830)	0.116
Enteral feeding begun before day 7	% (n/N)	87.5% (175/200)	82.2% (682/830)	0.070
Days to enteral feeding	Median (range)	3 (0–24) (n=200)	3 (0–34) (n=830)	0.006
Feeding difficulties at 3 years	% (n/N)	22.2% (34/153)	16.1% (68/423)	0.088
Severe neurodevelopment disability at 3 years	% (n/N)	10.5% (16/153)	14.4% (61/423)	0.217

*1031 children survived to 3 years, among which 200 children were assessed at 11 years.

IMD, Index of Multiple Deprivation.

#### Growth at 11 years

Growth data were available for 199 EP children (<27 weeks) and 143 controls. Mean z-scores for all growth measures apart from BMI in EP children were significantly below controls (table 2): Δ was −0.42 SD for weight, −0.45 SD for height and −1.05 SD for HC. These differences remained significant after adjusting for IMD at 11 years. For EP children, rates of overweight and obesity were 16.7% (33/198) and 4.0%, respectively, not significantly different from for controls (15.5% and 5.6%, respectively). In EP children, mean HC z-score decreased by 0.21 SD for each gestational week (95% CI 0.01 to 0.41; table 2).

**Table 2:** Growth parameters for EP children and controls at 11 years of age in the 2006 cohort

	EP: Mean (95% CI)					P value*	Controls: Mean (95% CI)	EP vs controls difference in means (95% CI)	P value
	≤23 weeks	24 weeks	25 weeks	26 weeks	All				
Height (cm)	149.9 (145.1 to 154.7) (n=14)	147.0 (143.4 to 150.5) (n=28)	149.8 (147.6 to 152.0) (n=69)	148.4 (146.1 to 150.6) (n=87)	148.8 (147.4 to 150.1) (n=198)	0.825	151.6 (150.3 to 152.8) (n=143)	−2.77 (−4.70 to −0.85)	0.005
Weight (kg)	46.3 (37.1 to 55.5) (n=15)	42.0 (38.3 to 45.8) (n=28)	42.1 (39.4 to 44.7) (n=69)	41.3 (38.9 to 43.6) (n=87)	42.0 (40.5 to 43.6) (n=199)	0.193	44.8 (43.0 to 46.6) (n=142)	−2.76 (−5.17 to −0.36)	0.024
BMI (kg/m^2^)	20.8 (17.9 to 23.6) (n=14)	19.3 (17.9 to 20.7) (n=28)	18.6 (17.7 to 19.5) (n=69)	18.5 (17.8 to 19.2) (n=87)	18.8 (18.3 to 19.3) (n=198)	0.056	19.4 (18.7 to 20.0) (n=142)	−0.57 (−1.37 to 0.23)	0.161
Head circumference (cm)	52.4 (51.0 to 53.8) (n=15)	52.4 (51.5 to 53.2) (n=28)	52.6 (52.0 to 53.2) (n=64)	52.8 (52.3 to 53.3) (n=87)	52.7 (52.3 to 53.0) (n=194)	0.190	54.1 (53.8 to 54.4) (n=142)	−1.47 (−1.93 to −1.01)	<0.001
Height z-score	0.26 (−0.37 to 0.89) (n=14)	−0.15 (−0.64 to 0.35) (n=28)	0.17 (−0.12 to 0.46) (n=69)	0.04 (−0.24 to 0.33) (n=87)	0.08 (−0.10 to 0.25) (n=198)	0.882	0.53 (0.37 to 0.68) (n=143)	−0.45 (−0.70 to −0.20)	<0.001
Weight z-score	0.60 (−0.22 to 1.42) (n=15)	0.24 (−0.25 to 0.73) (n=28)	0.16 (−0.15 to 0.46) (n=69)	0.11 (−0.16 to 0.38) (n=87)	0.18 (0.00 to 0.36) (n=199)	0.334	0.61 (0.43 to 0.78) (n=142)	−0.42 (−0.68 to −0.17)	0.001
BMI z-score	0.91 (0.23 to 1.59) (n=14)	0.40 (−0.12 to 0.92) (n=28)	0.03 (−0.30 to 0.36) (n=69)	0.11 (−0.16 to 0.38) (n=87)	0.18 (−0.00 to 0.36) (n=198)	0.108	0.43 (0.24 to 0.63) (n=142)	−0.26 (−0.53 0.02)	0.065
Head circumference z-score	−1.57 (−2.44 to −0.70) (n=15)	−1.60 (−2.17 to −1.03) (n=28)	−1.56 (−1.87 to −1.25) (n=63)	−1.15 (−1.46 to −0.84) (n=85)	−1.38 (−1.58 to −1.18) (n=191)	0.040	−0.34 (−0.55 to −0.12) (n=142)	−1.05 (−1.35 to −0.75)	<0.001

*This refers to P values for the associations of gestational age with growth parameters using linear regression with gestational age in weeks and days.

EP, extremely preterm.

### Comparison of outcomes for births <26 weeks of gestation in EPICure and EPICure2

#### Comparative characteristics

In EPICure, 309 children survived to 2.5 years among which 260 were born to mothers resident in England and 176 were assessed at 11 years. In EPICure2, 584 children born <26 weeks’ gestation survived to 3 years and 112 were assessed at 11 years. EPICure2 children were more likely to have received breast milk, to have parenteral and enteral feeding begun earlier and to be born to older mothers (table 3); they were less likely to have received postnatal systemic steroids; there were more South Asian and Black children and fewer White children in EPICure2. At the 11-year assessment, children’s chronological ages were significantly higher in EPICure2. The two cohorts were evenly matched in birth weight, gestational age, sex, multiple birth and IMD at 11 years. Differences in perinatal and demographic characteristics were accounted for when comparing growth between the two cohorts.

**Table 3:** Representative status of children born <26 weeks of gestation in England from the EPICure cohorts at 11 years and comparative characteristics for the two cohorts

		EPICure 1995		EPICure2 2006		Children assessed at 11 years
		Whole sample survivors by 2.5 years	Children assessed at 11 years	Whole sample survivors by 3 years	Children assessed at 11 years	2006 vs 1995
		n=260	n=176	n=584	n=112	P value
Gestational age						
<24 weeks	% (n/N)	9.2% (24/260)	10.8% (19/176)	11.3% (66/584)	13.4% (15/112)	0.337
24 weeks	% (n/N)	31.9% (83/260)	33.0% (58/176)	30.3% (177/584)	25.0% (28/112)	
25 weeks	% (n/N)	58.8% (153/260)	56.3% (99/176)	58.4% (341/584)	61.6% (69/112)	
Birth weight (g)	Mean (SD)	748.1 (109.9) (n=260)	745.8 (109.1) (n=176)	735.2 (120.3) (n=584)	739.6 (119.0) (n=112)	0.647
Birth weight z scores	Mean (SD)	−0.2 (0.8) (n=259)	−0.1 (0.8) (n=175)	−0.2 (0.7) (n=581)	−0.2 (0.7) (n=112)	0.391
Male sex	% (n/N)	48.8% (127/260)	45.5% (80/176)	47.9% (280/584)	50.0% (56/112)	0.451
Multiple birth	% (n/N)	25.8% (67/260)	29.0% (51/176)	22.1% (129/584)	24.1% (27/112)	0.365
Ethnicity						
White	% (n/N)	73.4% (190/259)	78.9% (138/175)	62.9% (363/577)	55.5% (61/110)	<0.001
Asian	% (n/N)	7.3% (19/259)	5.7% (10/175)	10.6% (61/577)	19.1% (21/110)	
Black	% (n/N)	17.4% (45/259)	14.9% (26/175)	22.0% (127/577)	20.9% (23/110)	
Other	% (n/N)	1.9% (5/259)	0.6% (1/175)	4.5% (26/577)	4.5% (5/110)	
Maternal age at delivery	Mean (SD)	28.5 (5.9) (n=259)	28.8 (5.7) (n=175)	29.4 (6.4) (n=584)	30.8 (5.7) (n=112)	0.006
Breast milk at any time	% (n/N)	85.0% (221/260)	86.4% (152/176)	96.1% (560/583)	100.0% (112/112)	<0.001
Parenteral nutrition- amino acids begun before day 7	% (n/N)	95.6% (238/249)	95.2% (160/168)	99.7% (581/583)	100.0% (111/111)	0.024
Days to amino acids after birth	Median (range)	3 (0–32) (n=249)	3 (0–32) (n=168)	2 (0–14) (n=583)	2 (0–14) (n=111)	<0.001
Parenteral nutrition- lipids begun before day 7	% (n/N)	85.1% (200/235)	86.3% (138/160)	98.3% (568/578)	99.1% (110/111)	<0.001
Days to lipids after birth	Median (range)	3 (0–33) (n=235)	3 (0–33) (n=160)	2 (0–14) (n=578)	2 (0–8) (n=111)	<0.001
Enteral feeding begun before day 7	% (n/N)	47.6% (121/254)	49.1% (84/171)	82.2% (480/584)	86.6% (97/112)	<0.001
Days to enteral feeding after birth	Median (range)	8 (2–43) (n=254)	8 (2–41) (n=171)	3 (0–31) (n=584)	3 (0–24) (n=112)	<0.001
Antenatal systemic steroids	% (n/N)	79.9% (207/259)	82.3% (144/175)	87.9% (509/579)	88.4% (99/112)	0.161
Postnatal systemic steroids	% (n/N)	71.4% (185/259)	71.4% (125/175)	20.4% (119/584)	24.1% (27/112)	<0.001
IMD at birth	Mean (SD)	–	–	4.2 (2.9) (n=581)	4.3 (2.7) (n=111)	–
IMD at 11 years	Mean (SD)	–	5.1 (2.8) (n=174)	–	4.9 (2.8) (n=111)	0.604
Age assessed at infancy	Mean (SD)	2.5 (0.1) (n=235)	2.5 (0.1) (n=171)	2.9 (0.4) (n=325)	2.9 (0.3) (n=88)	<0.001
Age assessed at 11 years	Mean (SD)	–	10.9 (0.4) (n=176)	–	11.9 (0.5) (n=112)	<0.001

IMD, Index of Multiple Deprivation.

#### Comparing growth between two cohorts

Growth measure z-scores were available for 111 EPICure2 children and 175 EPICure children. At 11 years, using repeated measures mixed models Δ between cohorts for weight was 0.72 SD (0.47 to 0.96), for height 0.55 SD (0.29 to 0.81) and for BMI 0.56 SD (0.24 to 0.87). These differences remained significant but reduced after adjustment for perinatal and demographic differences between eras (table 4). EPICure2 children had significantly lower rates of thinness compared with EPICure children (14% vs 27%; table 4). In contrast, Δ for HC z-scores was not significantly different in both cohorts and remained similar after adjusting for differences in maternal age, ethnicity, days to start parenteral nutrition and receipt of breast milk, but it became significant after further adjusting for differences in days to start enteral feeding and postnatal treatment with systemic steroids (Δ −0.46 SD (−0.83 to –0.10); table 4). At 11 years, 69% of EPICure2 children had HC z-scores of <–1 SD compared with 64% in EPICure (p=0.407).

**Table 4:** A comparison of growth measures between 1995 and 2006 for children born <26 weeks of gestation in England

			EPICure 1995	EPICure2 2006	2006 vs 1995	2006 vs 1995
			Mean (95% CI)	Mean (95% CI)	Unadjusted difference in means (95% CI)*	Adjusted difference in means (95% CI)*
*Birth*						
Weight z-score			−0.16 (−0.25 to −0.07) (n=265)	−0.25 (−0.31 to −0.19) (n=590)	−0.09 (−0.24 to 0.07)	−**0.26 (−0.45 to –0.07**)
Head circumference z-score			−0.23 (−0.42 to −0.05) (n=171)	−0.40 (−0.51 to −0.28) (n=291)	−0.17 (−0.43 to 0.04)	−**0.44 (−0.74 to –0.13**)
*EDD*						
Weight z-score			−1.73 (−1.86 to −1.60) (n=251)	−1.95 (−2.05 to −1.85) (n=581)	−**0.23 (−0.39 to –0.07**)	−**0.40 (−0.58 to –0.21**)
Head circumference z-score			−0.98 (−1.17 to −0.78) (n=244)	−1.29 (−1.43 to −1.16) (n=552)	−**0.33 (−0.54 to –0.12**)	−**0.60 (−0.86 to –0.34**)
*2.5–3 years* **†**						
Height z-score			−0.65 (−0.81 to −0.49) (n=216)	−0.44 (−0.60 to −0.27) (n=301)	0.21 (−0.00 to 0.43)	0.14 (−0.13 to 0.42)
Weight z-score			−1.20 (−1.37 to −1.03) (n=225)	−0.69 (−0.84 to −0.55) (n=316)	**0.49 (0.31 to 0.67**)	**0.30 (0.09 to 0.51**)
BMI z-score			−1.07 (−1.26 to −0.88) (n=211)	−0.48 (−0.63 to −0.33) (n=294)	**0.59 (0.36 to 0.83**)	**0.58 (0.28 to 0.88**)
Head circumference z-score			−1.65 (−1.84 to −1.46) (n=231)	−1.73 (−1.89 to −1.58) (n=316)	−0.13 (−0.37 to 0.11)	−**0.38 (−0.66 to –0.10**)
***11 years***						
Height z-score			−0.51 (−0.66 to −0.36) (n=175)	0.10 (−0.13 to 0.33) (n=111)	**0.55 (0.29 to 0.81**)	**0.43 (0.11 to 0.74**)
Weight z-score			−0.48 (−0.66 to −0.30) (n=174)	0.24 (−0.01 to 0.48) (n=112)	**0.72 (0.47 to 0.96**)	**0.51 (0.24 to 0.78**)
BMI z-score			−0.34 (−0.53 to −0.14) (n=174)	0.23 (−0.02 to 0.49) (n=111)	**0.56 (0.24 to 0.87**)	**0.50 (0.13 to 0.87**)
Head circumference z-score			−1.35 (−1.54 to −1.16) (n=174)	−1.57 (−1.83 to −1.31) (n=106)	−0.23 (−0.56 to 0.09)	−**0.46 (−0.83 to –0.10**)

	% (n/N)	% (n/N)	Overweight/obesity vs normal weight Unadjusted RRR (95% CI)‡	Thinness vs normal weight Unadjusted RRR (95% CI)‡	Overweight/obesity vs normal weight Adjusted RRR (95% CI)*	Thinness vs normal weight Adjusted RRR (95% CI)*
*Overweight/obesity at 2.5–3 years*						
Thinness	42.7% (90/211)	27.9% (84/301)	**1.48 (0.71 to 3.09**)	**0.54 (0.37 to 0.79**)	**2.85 (1.14 to 7.12**)	0.65 (0.39 to 1.10)
Normal weight	52.1% (110/211)	62.8% (189/301)				
Overweight	4.3% (9/211)	7.0% (21/301)				
Obesity	0.9% (2/211)	2.3% (7/301)				
*Overweight/obesity at 11 years*						
Thinness	27.0% (47/174)	14.4% (16/111)	1.31 (0.85 to 2.01)	**0.48 (0.29 to 0.79**)	2.10 (0.86 to 5.12)	0.59 (0.25 to 1.35)
Normal weight	61.5% (107/174)	62.2% (69/111)				
Overweight	10.9% (19/174)	18.0% (20/111)				
Obesity	0.6% (1/174)	5.4% (6/111)				

Bold fonts indicate significant differences between the two cohorts.

*Differences in mean z-scores for growth measures between the two cohorts at comparable ages were estimated using repeated measures mixed models. We adjusted for differences in perinatal and demographic characteristics, including maternal age at delivery, ethnicity (white, black, South Asian or other), days to start amino acids, days to start lipids, days to start enteral feeding, breast milk at any time (yes vs no), any postnatal systemic steroids (yes vs no).

†Growth measures corrected for prematurity at 2.5–3 years.

‡RRRs of child overweight/obesity and thinness for EPICure2 2006 compared with EPICure 1995 were estimated using multinomial logistic regression models.

EDD, expected date of delivery; RRR, relative risk ratio.

**Figure 1:**
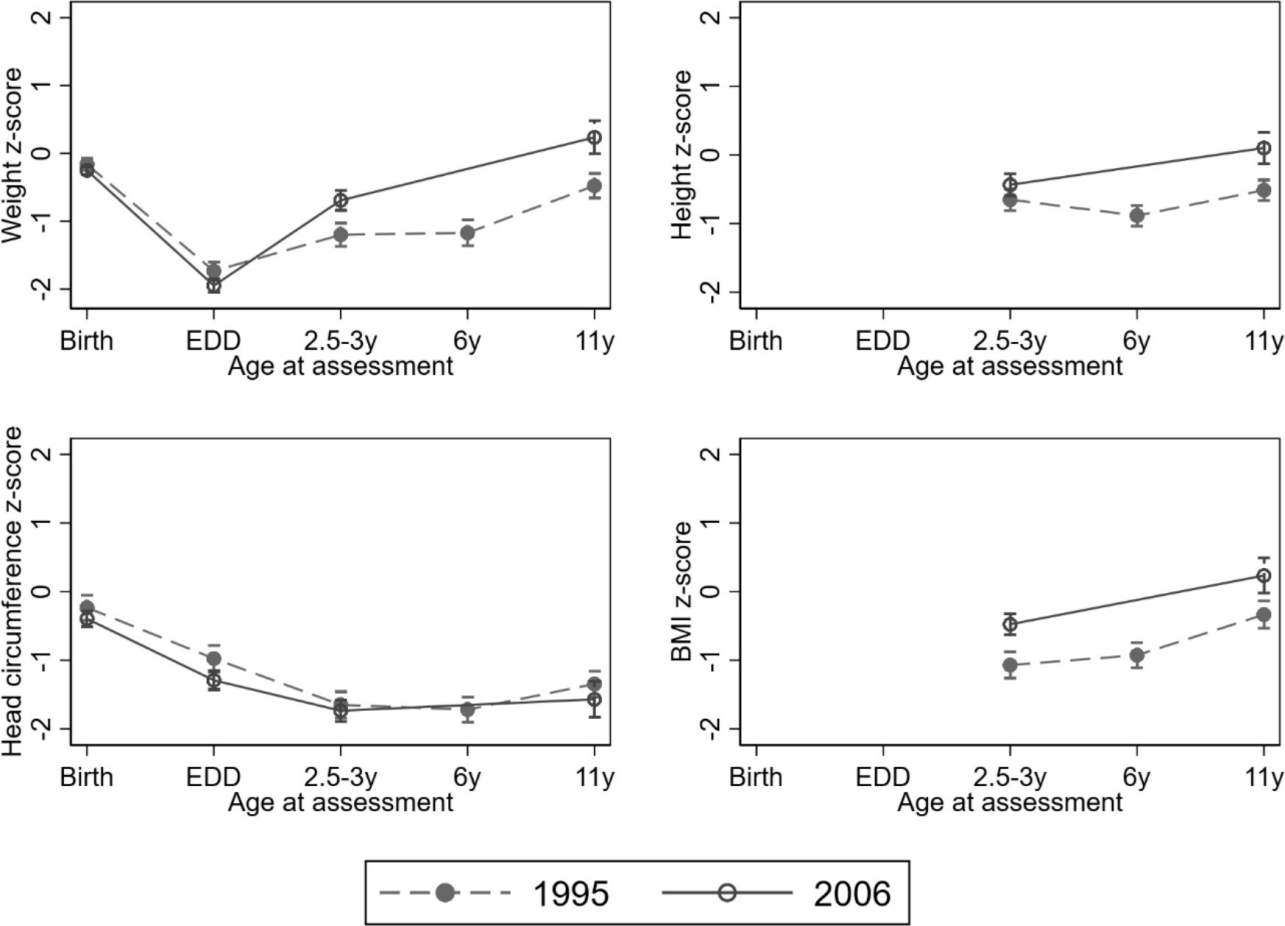
Observed means and 95% CIs in z-scores for growth parameters at different ages in 1995 (EPICure) and 2006 (EPICure2).

Using repeated measures mixed models the estimated postnatal fall in mean weight z-scores from birth to EDD was similar in both cohorts (figure 1). Both cohorts demonstrated catch-up in weight after EDD up to 3 years, but this was estimated to be greater in EPICure2 than in EPICure (EPICure2: 1.25 SD (1.12, 1.38) vs EPICure: 0.53 SD (0.37, 0.70); p<0.001). From 2.5–3 years to 11 years, improved growth was observed in EPICure2 compared with EPICure (figure 1). In contrast, HC from birth to 11 years of age was similar in both cohorts: significantly below population norms at all ages, a relative decline from birth to 2.5–3 years, followed by minimal catch-up (figure 1).

## Discussion

We have reported growth outcomes at 11 years of age for EP children and term-born controls in EPICure2 and compared growth outcomes from birth to age 11 years for children born <26 weeks’ gestation with EPICure. In EPICure2 at 11 years, mean growth in weight and length were comparable to the UK 1990 growth standards, but EP children were significantly shorter and lighter than controls at 11 years. Rates of overweight and obesity were similar in two groups. Compared with EPICure, improvements were observed in weight, height and BMI during infancy and middle childhood in EPICure2 and reassuringly the rate of thinness was lower in EPICure2 than in EPICure. Greater catch-up in weight from EDD to 2.5–3 years was observed in EPICure2 compared with EPICure. In contrast, head growth was poor during childhood in both studies.

Our data show that weight, height and BMI during infancy and middle childhood have improved for babies born <26 weeks of gestation in 2006 compared with births in 1995, contrasting with the Victorian Cohort which showed no improvement in growth across three eras: 1991–1992, 1997 and 2005.^18^ In our study, improvements were partly explained by known perinatal and demographic differences between eras. This might also partly reflect secular trends in human physical growth,^19 20^ as this was also shown in controls (online supplemental table S3). Secular changes are directly linked to improved access to health and nutrition and also correlated with education, income and social status and infection.^20^ Moreover, secular gains in weight and BMI may reflect reductions in physical activity associated with an increasingly sedentary lifestyle.^21^

Greater catch-up in weight from EDD to 2.5–3 years occurred in EPICure2 than in EPICure. This may be of concern, because evidence from both term and preterm populations shows an association of rapid catch-up growth in infancy and early childhood with increased risk for cardiovascular disease and type 2 diabetes in adulthood.^22–27^ In particular, we have also shown that EP young adults with metabolic syndrome at 19 years tended to have greater catch-up in weight from EDD to 2.5 years compared with those without metabolic syndrome.^28^

Our comparison of the two cohorts shows that, despite improvements in perinatal care, there was no improvement in head growth: after full adjustment, EPICure2 children had heads 0.46 SD smaller than the earlier cohort. Brain development begins in the second trimester of pregnancy and continues into adult life, and multiple complex events critical for brain development occur between 24 and 40 weeks’ gestation.^29 30^ Thus, lower gestation at birth is associated with smaller brain volume at birth and in the early postnatal period^31 32^ and positive associations of postnatal head growth with neurocognitive outcomes have been demonstrated at ages ranging from infancy to adulthood in preterm populations.^33 34^ The lack of improvement in head growth may reflect the lack of change in neurodevelopmental outcomes between the two cohorts.^12^ Interestingly, we also observed an unexplained decrease in head size in controls (−0.32 SD; online supplemental table S3).

The strengths of this paper include comparing identical growth measures across two cohorts using common standards, applying multiple imputation to account for missing data and recruiting a contemporary comparison group for each. The major limitation in EPICure2 is the small number of participants seen at 11 years representing 41.5% (200/482) of the chosen sample; thus, the proportion lost to follow-up increased over time, as shown in EPICure.^4^ However, drop-out analysis showed that children assessed at 11 years were representative of the original cohort in baseline characteristics, and our findings were strengthened by using multiple imputation. Further, controls were recruited from mainstream schools and might be relatively more healthy than the general population. Data from the National Child Measurement Programme for England show that the prevalence of obesity for children aged 10–11 was 20% in 2017/2018 in England. Thus, group differences in BMI and rates of overweight/obesity may have been underestimated in this study. It is also possible that group difference in height may have been overestimated. Additionally, although the two cohorts were comparable in birth characteristics and IMD at 11 years, EPICure2 EP children were on average 1 year older than those in EPICure. However, we used z-scores based on UK norms which were adjusted for age and sex. Last, significant differences were found in other perinatal and demographic characteristics between cohorts, but they were adjusted for in the models and did not fully explain differences in somatic growth over time.

Despite advancing perinatal care and survival since 1995, head growth has not improved for children born <26 weeks’ gestation in 2006. No improvement in brain volume development may indicate similar neurocognitive development in the two cohorts. Early head growth failure could be improved by increasing breast milk feeding^35^ or parenteral nutrition protein and calorie intake in early life.^36 37^ In contrast, we have shown improvements in somatic growth during infancy and middle childhood among EPICure2 children, who have greater weight catch-up in infancy, that is maintained. By age 11, EPICure2 EP children do remain shorter and lighter than term-born controls, but rates of overweight and obesity were similar and growth attainment in weight and height was not different from the population norms used. Growth in childhood remains an important issue in preterm populations and new strategies to improve head growth in particular across childhood are urgently needed. It is important to alert parents/carers and primary healthcare professionals of the increasing evidence of metabolic risk from being overweight/obese for EP children.

## Data Availability

Data are available on reasonable request. Data are available subject to the EPICure Data Sharing Policy (www.epicure.ac.uk) and will be available as part of the RECAP preterm Cohort Platform (https://recap-preterm.eu)
