# Supplementary material for: Growth in extremely preterm children born in England in 1995 and 2006: the EPICure studies
Source: Arch Dis Child Fetal Neonatal Ed. 2021 Jul 13;107(2):193–200. doi: 10.1136/archdischild-2020-321107 (PMC8867260; doi:10.1136/archdischild-2020-321107)
Supplement: Supplementary data [file fetalneonatal-2020-321107supp001.pdf]

Supplementary Appendix

Methods

Assessment All children assessed at 11 years in the EPICure2 study were evaluated by a trained clinical assessor and a psychologist who travelled to the child's school or home and conducted comprehensive physical and neuropsychological evaluations. Height was measured using a portable height measure. Weight was measured using a calibrated scale (SECA 803). HC was measured using a non-distensible Lasso-o tape. The tape was placed above the ears and midway between the eyebrows and the hairline to the occipital prominence at the back of the head. The aim was to always measure the largest circumference possible. The tape was pulled so that any hair was compressed. Three readings were taken for weight, height and HC, and overall means were calculated for each. Body mass index (BMI) was calculated as weight in kilograms divided by height in metres squared.

Supplementary Tables

Table S1 Variables used for multiple imputations, type of variable, model used to predict missing data, and percentages of values missing for each variable included in the imputation model. Missing data were imputed by chained equations using the STATA "MI" procedure. Imputation models were based on the missing at random assumption. We created twenty imputed datasets.

Cohort	Variable	Type of variable	Model used to predict missing data	Percentages of values missing
**2006**				
	Birth weight	Continuous	No missing data	0%
	Gestational age	Continuous	No missing data	0%
	Sex	Binary	No missing data	0%
	Multiple birth	Binary	No missing data	0%
	Maternal age at delivery	Continuous	Linear regression	0.1% (1/1031)
	Breast milk at discharge	Binary	Binary logistic regression	0.3% (3/1031)
	Any antenatal steroids	Binary	Binary logistic regression	1.2% (12/1031)
	Enteral feeding before day 7	Binary	Binary logistic regression	0.1% (1/1031)
	Maternal smoking during pregnancy	Binary	Binary logistic regression	5.5% (57/1031)
	Worst cerebral ultrasound scan	Binary	Binary logistic regression	0.5% (5/1031)
	IMD at birth	Continuous	Linear regression	0.9% (9/1031)
	Maternal height	Continuous	Linear regression	19.4% (200/1031)
	Maternal weight	Continuous	Linear regression	21.2% (219/1031)
	Severe neurodevelopment disability at 3y	Binary	Binary logistic regression	44.1% (455/1031)
	IMD at 11y	Continuous	Linear regression	81.1% (836/1031)
	Weight at expected date of delivery	Continuous	Linear regression	1.6% (16/1031)
	Head circumference at birth	Continuous	Linear regression	51.8% (534/1031)
	Head circumference at expected date of delivery	Continuous	Linear regression	7.2% (74/1031)
	Height at 3y	Continuous	Linear regression	47.2% (487/1031)
	Weight at 3y	Continuous	Linear regression	45.3% (467/1031)
	BMI at 3y	Continuous	Linear regression	47.8% (493/1031)
	Head circumference at 3y	Continuous	Linear regression	45.0% (464/1031)
	Height at 11y	Continuous	Linear regression	80.8% (833/1031)
	Weight at 11y	Continuous	Linear regression	80.7% (832/1031)
	BMI at 11y	Continuous	Linear regression	80.8% (833/1031)
	Head circumference at 11y	Continuous	Linear regression	81.2% (837/1031)
**1995**				
	Birth weight	Continuous	No missing data	0%
	Gestational age	Continuous	No missing data	0%
	White ethnicity	Binary	No missing data	0%
	Sex	Binary	No missing data	0%
	Severe changes of respiratory distress syndrome	Binary	No missing data	0%
	Necrotising enterocolitis	Binary	No missing data	0%
	Breast milk at any time	Binary	No missing data	0%
	Bronchopulmonary dysplasia	Binary	No missing data	0%
	Enteral feeding before day 7	Binary	Binary logistic regression	2.6% (8/309)
	Any antenatal steroids	Binary	Binary logistic regression	0.6% (2/309)
	Severe neurodevelopment disability at 2.5y	Binary	Binary logistic regression	8.4% (26/309)
	Socioeconomic status at 2.5y	Three-category	Ordinal logistic regression	12.3% (38/309)
	IMD at 11y	Continuous	Linear regression	43.4% (134/309)
	Weight at expected date of delivery	Continuous	Linear regression	2.6% (8/309)
	Head circumference at birth	Continuous	Linear regression	33.0% (102/309)
	Head circumference at expected date of delivery	Continuous	Linear regression	6.8% (21/309)
	Height at 2.5y	Continuous	Linear regression	16.5% (51/309)
	Weight at 2.5y	Continuous	Linear regression	12.3% (38/309)
	BMI at 2.5y	Continuous	Linear regression	18.1% (56/309)
	Head circumference at 2.5y	Continuous	Linear regression	10.0% (31/309)
	Height at 6y	Continuous	Linear regression	23.3% (72/309)
	Weight at 6y	Continuous	Linear regression	22.0% (68/309)
	BMI at 6y	Continuous	Linear regression	23.3% (72/309)
	Head circumference at 6y	Continuous	Linear regression	22.7% (70/309)
	Height at 11y	Continuous	Linear regression	29.4% (91/309)
	Weight at 11y	Continuous	Linear regression	29.8% (92/309)
	BMI at 11y	Continuous	Linear regression	29.8% (92/309)
	Head circumference at 11y	Continuous	Linear regression	29.8% (92/309)

All variables were included in the linear predictor of all imputation models. N=1031 survivors at 3 years for EPICure 2006; N=309 survivors at 2.5 years for EPICure 1995.

Table S2 Original and imputed growth measures for births between 22 and 25 weeks of gestation in England in the 1995 and 2006 cohorts

(a)	1995 Original					1995 Imputed: 20 imputed datasets (N=260)				
	Birth	EDD	2.5y	6y	11y	Birth	EDD	2.5y	6y	11y
Height (cm)	-	-	88.7 (88.1, 89.2) [n=216]	112.7 (111.9, 113.6) [n=194]	139.6 (138.6, 140.7) [n=175]	-	-	88.7 (88.1, 89.3)	113.1 (112.3,114.0)	140.2 (139.1,141.4)
Weight (kg)	0.75 (0.73, 0.76) [n=266]	2.55 (2.49, 2.61) [n=258]	11.8 (11.6, 12.1) [n=225]	18.7 (18.2, 19.3) [n=198]	33.4 (32.3, 34.5) [n=174]	0.75 (0.73, 0.76)	2.56 (2.50, 2.62)	11.9 (11.7, 12.1)	18.9 (18.3, 19.4)	34.4 (33.1, 35.7)
BMI (kg/m^2)	-	-	15.1 (14.9, 15.3) [n=211]	14.5 (14.3, 14.8) [n=194]	17.0 (16.6, 17.4) [n=174]	-	-	15.1 (14.9, 15.3)	14.7 (14.4, 15.0)	17.3 (16.9, 17.8)
Head circumference (cm)	23.3 (23.0, 23.5) [n=172]	33.2 (32.9, 33.5) [n=250]	48.2 (48.0, 48.5) [n=231]	50.4 (50.2, 50.7) [n=196]	52.4 (52.1, 52.7) [n=174]	23.2 (23.0, 23.5)	33.2 (33.0, 33.5)	48.2 (48.0, 48.5)	50.5 (50.2, 50.7)	52.5 (52.2, 52.7)

(b)	2006 Original				2006 Imputed: 20 imputed datasets (N=584)			
	Birth	EDD	3y	11y	Birth	EDD	3y	11y
Height (cm)	-	-	92.9 (92.2, 93.6) [n=304]	149.1 (147.4, 150.8) [n=111]	-	-	92.6 (92.0, 93.3)	148.7 (147.2,150.2)
Weight (kg)	0.73 (0.72, 0.74) [n=593]	2.54 (2.49, 2.60) [n=582]	13.4 (13.2, 13.7) [n=318]	42.6 (40.5, 44.8) [n=112]	0.74 (0.73, 0.75)	2.55 (2.50, 2.60)	13.4 (13.1, 13.7)	43.4 (41.4, 45.4)
BMI (kg/m^2)	-	-	15.6 (15.3, 15.8) [n=301]	19.0 (18.3, 19.8) [n=111]	-	-	15.6 (15.3, 15.8)	19.4 (18.8, 20.0)
Head circumference (cm)	23.1 (22.9, 23.2) [n=292]	32.9 (32.7, 33.1) [n=553]	48.5 (48.3, 48.7) [n=320]	52.5 (52.1, 52.9) [n=107]	23.0 (22.8, 23.1)	33.0 (32.8, 33.2)	48.3 (48.1, 48.5)	52.6 (52.2, 53.1)

Table S3 Growth measures at 11 years for births between 22 and 25 weeks of gestation and term-born controls in England in the 1995 and 2006 cohorts

	EP 1995 at 11y		EP 2006 at 11y		1995 EP vs Controls		2006 EP vs Controls		Controls 2006 vs 1995	
	EP Mean (SD)	Controls Mean (SD)	EP Mean (SD)	Controls Mean (SD)	Difference in means (95%CI)	*p*	Difference in means (95%CI)	*p*	Difference in means (95%CI)	*p*
Height (cm)	139.6 (7.0) [n=175]	144.5 (7.8) [n=153]	149.1 (9.1) [n=111]	151.6 (7.6) [n=143]	-4.91 (-6.52, -3.31)	<0.001	-2.47 (-4.53, -0.41)	0.019	7.04 (5.28, 8.79)	<0.001
Weight (kg)	33.4 (7.3) [n=174]	38.4 (9.9) [n=152]	42.6 (11.5) [n=112]	44.8 (11.0) [n=142]	-5.01 (-6.89, -3.12)	<0.001	-2.17 (-4.96, 0.62)	0.128	6.41 (4.01, 8.81)	<0.001
BMI (kg/m^2)	17.0 (2.7) [n=174]	18.2 (3.4) [n=152]	19.0 (3.9) [n=111]	19.4 (3.8) [n=142]	-1.22 (-1.89, -0.54)	<0.001	-0.33 (-1.29, 0.63)	0.494	1.18 (0.35, 2.01)	0.005
Head circumference (cm)	52.4 (1.7) [n=174]	54.3 (1.5) [n=152]	52.5 (2.2) [n=107]	54.1 (1.8) [n=142]	-1.92 (-2.27, -1.56)	<0.001	-1.61 (-2.12, -1.10)	<0.001	-0.19 (-0.57, 0.19)	0.320
Height z-score	-0.5 (1.0) [n=175]	0.2 (1.0) [n=153]	0.1 (1.2) [n=111]	0.5 (0.9) [n=143]	-0.67 (-0.89, -0.45)	<0.001	-0.42 (-0.69, -0.16)	0.002	0.37 (0.15, 0.60)	0.001
weight z-score	-0.5 (1.2) [n=174]	0.2 (1.2) [n=152]	0.2 (1.3) [n=112]	0.6 (1.0) [n=142]	-0.68 (-0.94, -0.43)	<0.001	-0.37 (-0.66, -0.08)	0.012	0.40 (0.14, 0.65)	0.002
BMI z-score	-0.3 (1.3) [n=174]	0.2 (1.3) [n=152]	0.2 (1.4) [n=111]	0.4 (1.2) [n=142]	-0.50 (-0.79, -0.22)	0.001	-0.20 (-0.51, 0.11)	0.208	0.27 (-0.01, 0.54)	0.062
Head circumference z-score	-1.3 (1.3) [n=174]	-0.0 (1.0) [n=152]	-1.6 (1.3) [n=106]	-0.3 (1.3) [n=142]	-1.33 (-1.58, -1.08)	<0.001	-1.23 (-1.57, -0.90)	<0.001	-0.32 (-0.59, -0.05)	<0.001
